# A load of mice to hypergravity causes AMPKα repression with liver injury, which is overcome by preconditioning loads via Nrf2

**DOI:** 10.1038/srep15643

**Published:** 2015-10-23

**Authors:** Sang Gil Lee, Chan Gyu Lee, Hong Min Wu, Choong Sik Oh, So Won Chung, Sang Geon Kim

**Affiliations:** 1 College of Pharmacy and Research Institute of Pharmaceutical Sciences, Seoul National University, Seoul, Korea; 2 Aerospace Medical Center, ROKAF, Cheong-ju, Korea

## Abstract

An understanding of the effects of hypergravity on energy homeostasis is necessary in managing proper physiological countermeasures for aerospace missions. This study investigated whether a single or multiple load(s) of mice to hypergravity has an effect on molecules associated with energy metabolism. In the liver, AMPKα level and its signaling were repressed 6 h after a load to +9 Gz hypergravity for 1 h, and then gradually returned toward normal. AMPKα level was restored after 3 loads to +9 Gz, suggestive of preconditioning adaptation. In cDNA microarray analyses, 221 genes were differentially expressed by +9 Gz, and the down-regulated genes included Nrf2 targets. Nrf2 gene knockout abrogated the recovery of AMPKα elicited by 3 loads to +9 Gz, indicating that Nrf2 plays a role in the adaptive increase of AMPKα. In addition, +9 Gz stress decreased STAT3, FOXO1/3 and CREB levels, which was attenuated during the resting time. Similarly, apoptotic markers were enhanced in the liver, indicating that the liver may be vulnerable to hypergravity stress. Preconditioning loads prevented hepatocyte apoptosis. Overall, a load of mice to +9 Gz hypergravity causes AMPKα repression with liver injury, which may be overcome by multiple loads to hypergravity as mediated by Nrf2.

Humans are adapted to live the accelerative force of gravity. Alteration of gravity (hypergravity and/or microgravity) represents a powerful physical cue to exert modeling effects on both anatomy and function of living organisms^1^,^2^,^3^,^4^,^5^,^6^,^7^,^8^,^9^. Hypergravity forces intermittently subject an organism to a myriad of stresses, potentially disrupting cytoskeletal and organelle structures, and energy metabolism. Exposure to altered hypergravity implies detrimental effects on muscle mass^10^, composition^11^, and contractility^3^, and on bone density, with long-term effects even after return to normal gravity^12^. Many studies have focused on simulated microgravity which had effects on the nervous system, in some cases not perturbing cell differentiation and assembling^13^,^14^,^15^, in other cases strongly altering cell morphology and functions^16^ or improving stem cell differentiation into neurons^8^,^17^,^18^.

Few reports deal with hypergravity effects on biological systems. Astronauts experience a hypergravity of +3.2 Gz at launch and +1.4 Gz on reentry. In case of manned spacecraft, astronauts were exposed up to +3.6 ~ +4.0 Gz for ~30 seconds when first engines cut-off after launch. They were then exposed to hypergravity up to +2 Gz for ~100 seconds when second engines cut-off^19^. Pilots of jet fighter plane may be exposed up to +9 Gz for a few seconds. It is generally accepted that a high gravitational acceleration force acting along +Gz causes considerable strain on organs. Thus, an understanding of the effects of hypergravity on energy homeostasis would be of help in managing proper countermeasures to assure safe and effective aerospace missions and human survival under a hypergravity condition. Exposure to hypergravity could induce protein ubiquitination of muscle cell line, demonstrating that ubiquitination was induced by treatment of the cells at +100 G for 1 h^20^. In addition, hypergravity affects myoblast proliferation and differentiation^21^, PC12 cell differentiation *in vitro*^22^, and cyclooxygenase-2 expression in the heart vessels *in vivo*^23^. Dysregulation of immune function by chronic exposure has also been reported^24^. In the study of Oguro *et al.*^25^, the expression of HSP47, a collagen-specific molecular chaperone, responds to gravitational changes (i.e., hypergravity or microgravity *in vitro* and *in vivo*).

The liver is a continuous sponge-like parenchymal mass consisting of the interdigitating networks of afferent and efferent vessels and is featured by compliance, high capacity and energy metabolism. Hence, the liver tissue may be more susceptible to hemodynamic changes such as physical or compressional stress. In addition to the direct pressure changes on the hepatic cell during aerospace craft launch or take-off and the accompanying temporal changes in physical integrity of cellular organelles, altered hemodynamic circulation and sheer stress would have an effect on macromolecules associated with metabolism in the liver and consequently energy production. At the molecular level, AMP-activated protein kinase (AMPK), an intracellular energy sensor, plays a key role in the regulation of transcription factors such as STAT3, FoxO1/3 and CREB, inhibiting hepatic gluconeogenesis and synthesis of glycogen, fatty acid and cholesterol^26^,^27^,^28^,^29^,^30^.

In view of the lack of an understanding on the effect of hypergravity stress and the impact of accompanying changes in hemodynamic circulation and sheer stress on biological system, this study investigated whether a single or multiple load(s) to +3 Gz or +9 Gz hypergravity has a detrimental effect on the integrity of the liver, skeletal muscle and kidney in a mouse model, and the molecules associated with energy metabolism, and cell viability, particular focusing on those associated with energy homeostasis in the liver. Here, we report that a single load of mice to +9 Gz causes a compressional injury stress on the liver, decreasing AMPKα and key transcription factor levels, reflective of liver susceptibility to hypergravity, and that the changes gradually return to basal conditions during resting times. Our finding also demonstrates the preventive effect of repetitive training loads on hypergravity stress and the role of nuclear factor erythroid-2-related factor 2 (Nrf2), a master transcription factor regulating cellular redox homeostasis, in the adaptation process.

## Results

### Effects of +3 or +9 Gz hypergravity load on tissue injury markers

**Figure 1:**
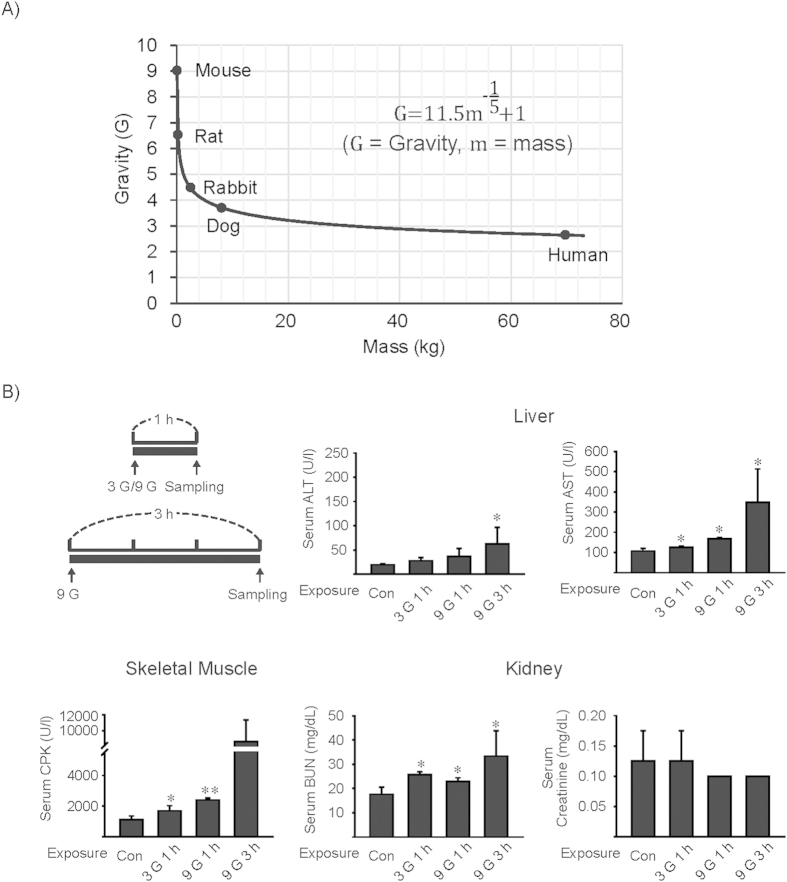
Changes of hematological indexes by a load to +3 or +9 Gz hypergravity. **(A)** A new equation created using reported effects of gravity on mortality vs. masses of different animals. A scaling in hypergravity was extrapolated to that of human using the proposed equation. **(B)** Hematological parameters. The activities of aminotransferases, creatine phosphate kinase (CPK), blood urea nitrogen (BUN) and creatinine contents in serum were measured as injury markers of the liver, skeletal muscle and kidney, respectively, immediately following exposure of mice to a single load to +3 or +9 Gz for 1 h or 3 h. Data represent the mean±S.E (N = 3-4 each). Statistical significance of the differences between hypergravity stress and control group (*P < 0.05, **P < 0.01). Con, control.

The relationship between the size of animal and its gravity response has been considered with the primary end point being survival, and scaling in hypergravity has been attempted in observations of the effects to animals. In the earlier work, a decrease in the magnitude of gravity load that an animal can survive was relative to its body mass among different species including mice, rats, rabbit and dogs^31^. In the current study, we created a new equation using reported effects of gravity on mortality vs. masses of the animal species, trying to extrapolate the data to human cases (Fig. 1A). The equation enabled us to predict gravity load in mice corresponding to humans; +9 Gz to mice approximated to the intensity of +2.7 G to humans.

To assess the effects of hypergravity forces on major organs, we first measured the activities of aminotransferases, creatine phosphate kinase (CPK), blood urea nitrogen (BUN) and creatinine contents in serum as representative injury markers of the liver, skeletal muscle and kidney, respectively, immediately after an exposure of mice to +3 or +9 Gz hypergravity (Fig. 1B). A load of mice to either +3 Gz or +9 Gz for 1 h significantly increased serum AST activity. There was also a tendency of increase in serum ALT activity. A load to either +3 or +9 Gz for 1 h significantly increased CPK and BUN in serum. Although exposure of mice to +9 Gz for 3 h increased both ALT and AST activities, two out of six animals died after treatment. In addition, a load to +9 Gz for 3 h increased BUN, but not in CPK, probably due to its smaller sample size (as a result of mortality). Our data raised the possibility that an exposure of mice to +3 or +9 Gz force for 1 h or 3 h may cause compressional injuries of the organs. In another set of experiments, we found increases of serum aminotransferase activities during the resting times following a load to +9 Gz for 1 h although other parameters such as CPK and BUN returned toward normal (data not shown). Subsequently, we chose +9 Gz for 1 h treatment regimen to acquire a non-lethal maximal effect. Since the liver seems to be more vulnerable to the stress of hypergravity load due to its sponge-like structural mass, we next focused on hepatic molecules associated with energy metabolism and cell viability.

### Alteration of AMPK signaling by +9 Gz hypergravity

**Figure 2:**
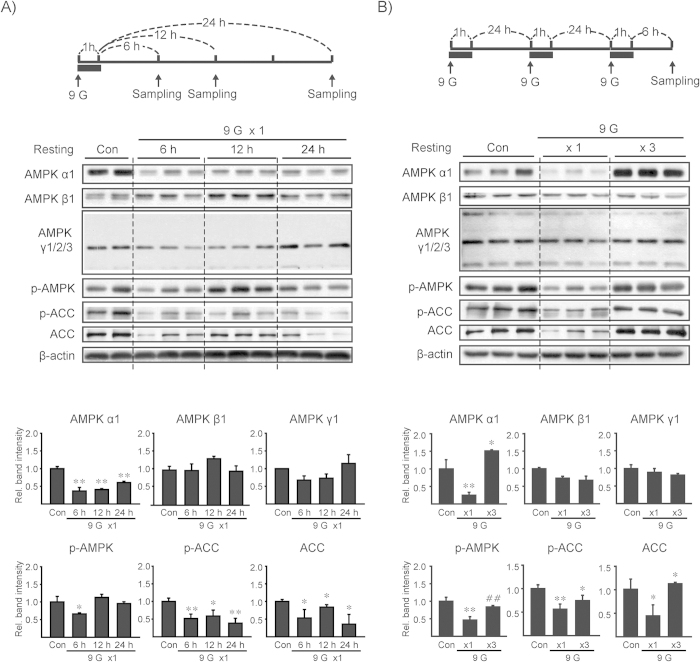
AMPK subunit levels and its signal intensity after load(s) to +9 Gz hypergravity. **(A, B**) Immunoblottings for AMPKα1, β1, γ1/2/3, p-AMPK, p-ACC and ACC in liver homogenates (Top). C57BL/6 mice were subjected to a single load to +1 Gz (control, Con) or +9 Gz for 1 h (x1), or to three consecutive daily loads (x3), and the liver samples were obtained at the indicated times after treatment. Of six different samples, two or three representative lanes were shown in each group. Scanning densitometry was done to assess relative changes (bottom). Data represent the mean±S.E (N = 6 each). Statistical significance of the differences was determined as compared to control group (*P < 0.05, **P < 0.01) or to a single load to +9 Gz (^##^*P* < 0.01).

Hypergravity load may trigger a cascade of molecular changes, inducing adaptation at later times. Next, we examined the status of AMPK, an intracellular sensor for energy homeostasis, by monitoring AMPK subunits and the signaling pathway 6, 12, or 24 h after a single load to +9 Gz hypergravity for 1 h. AMPKα levels were notably diminished 6 h after treatment, which returned toward normal at later times (Fig. 2A). In contrast, AMPKβ1 and γ1/2/3 were not notably changed. Consistently, the level of p-AMPK, an indicator of AMPK activity, was decreased 6 h after +9 Gz load, and this effect was attenuated at later times. p-ACC levels were maximally lowered 6 h after hypergravity stress, confirmative of the inhibition of AMPK. ACC levels were also diminished and then returned to normal condition. Hepatic AMPK signaling (p-AMPKα) was unchanged immediately after +9 Gz load (data not shown), implying that AMPK signaling might be diminished and recover during resting time.

We were also interested in the effect of multiple loads to +9 Gz hypergravity on AMPK subunits. In this model, a 6 h time-point after last exposure to +9 Gz was chosen to compare the effect with that of a single load. Interestingly, AMPKα level was almost completely restored 6 h after 3 consecutive daily loads to +9 Gz (Fig. 2B). p-ACC levels matched with this result. AMPK β1 and γ1/2/3 levels were comparable to those in control. Collectively, a single load of mice to +9 Gz hypergravity for 1 h suppressed AMPKα, but not other subunits, at 6 h after treatment, and this inhibition of AMPKα was overcome by repetitive preconditioning loads.

### cDNA microarray analysis of the genes affected by +9 Gz load

**Figure 3:**
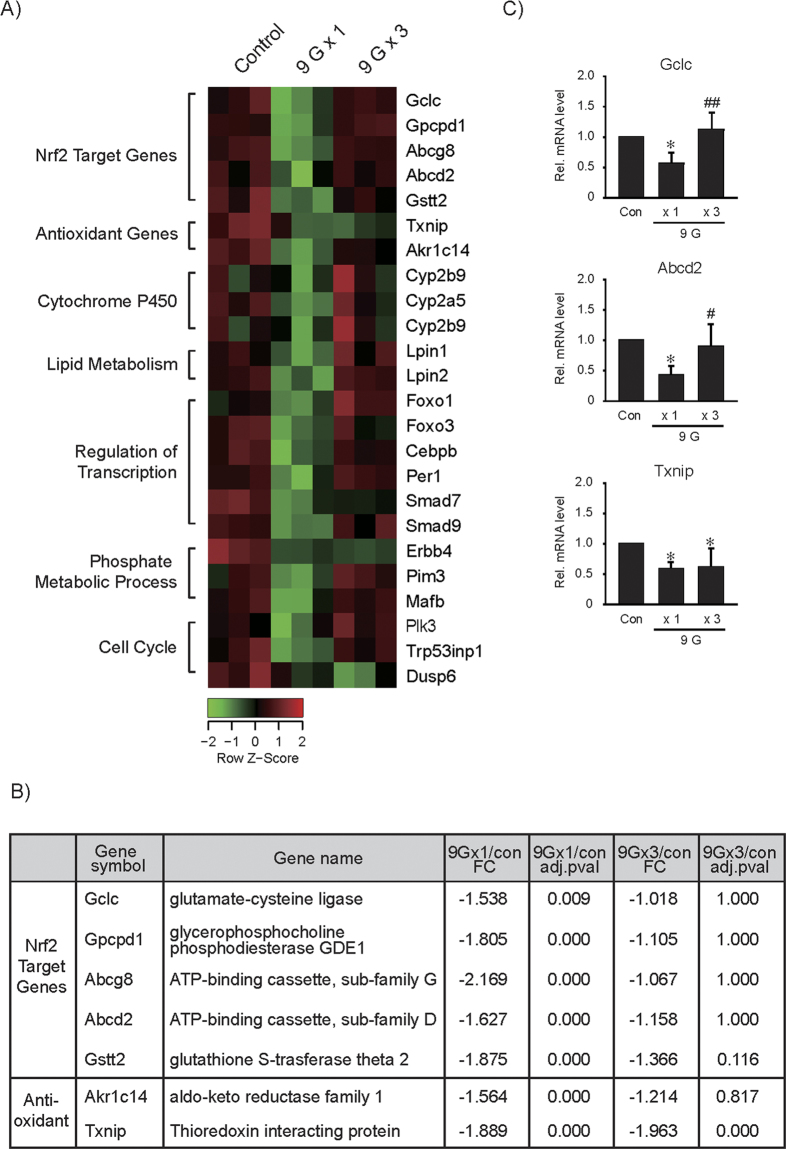
cDNA microarray and qRT-PCR assays. (**A**) Heat map. cDNA microarray analyses were done on RNA samples extracted from the liver. Mice were subjected to a single load to +1 Gz (control, Con) or +9 Gz for 1 h, or to three consecutive daily loads, and the liver samples were obtained 6 h after treatment (N = 3 each). (**B**) Relative changes and significance values for the transcripts of a subset of genes targeted by Nrf2 and two Nrf2-independent antioxidant genes. (**C**) qRT-PCR assays for Gclc, Abcd2 and Txnip in the liver. Data represent the mean±S.E (N = 4 each). Statistical significance of the differences was determined as compared to control group (*P < 0.05) or to a single load to +9 Gz treatment (^#^*P* < 0.05, ^##^*P* < 0.01).

As an effort to find out the genes whose expression is affected by hypergravity load(s), we performed cDNA microarray assays using the liver of mice exposed to a single or multiple load(s) of hypergravity (6 h after +9 Gz treatment for 1 h), and the microarray profiles were compared (Fig. 3A). Among those represented on the microarray, 221 genes were differentially expressed by a load of +9 Gz when *P* < *0.05* and a 1.5-fold change cutoff were used. Bioinformatic analyses using Functional Enrichment Analysis allowed us to select the genes down-regulated by a single load of hypergravity, which included Nrf2 target genes, antioxidant genes, cytochrome P450s, and the genes associated with lipid metabolism, transcriptional regulation, or cell cycle control (Fig. 3B). In DEG analysis, a load of +9 Gz suppressed a subset of Nrf2-target genes (Gclc, Gpcpd1, Abcg8, Abcd2 and Gstt2) and two other antioxidant genes. Nrf2 target genes were all significantly inhibited by a single exposure to hypergravity and were restored after multiple exposures. Of the antioxidant genes, *Akr1c14* mRNA level was comparable to that of Nrf2 target transcripts. In contrast, *Txnip* mRNA level, which was initially suppressed by a load to +9 Gz, failed to return toward basal after three loads to +9 Gz. qRT-PCR assays verified substantial and significant inhibition of Nrf2 target transcripts (Gclc and Abcd2), and this effect was abolished after 3 consecutive loads (Fig. 3C). The lack of adaptive *Txnip* recovery was also confirmed.

**Figure 4:**
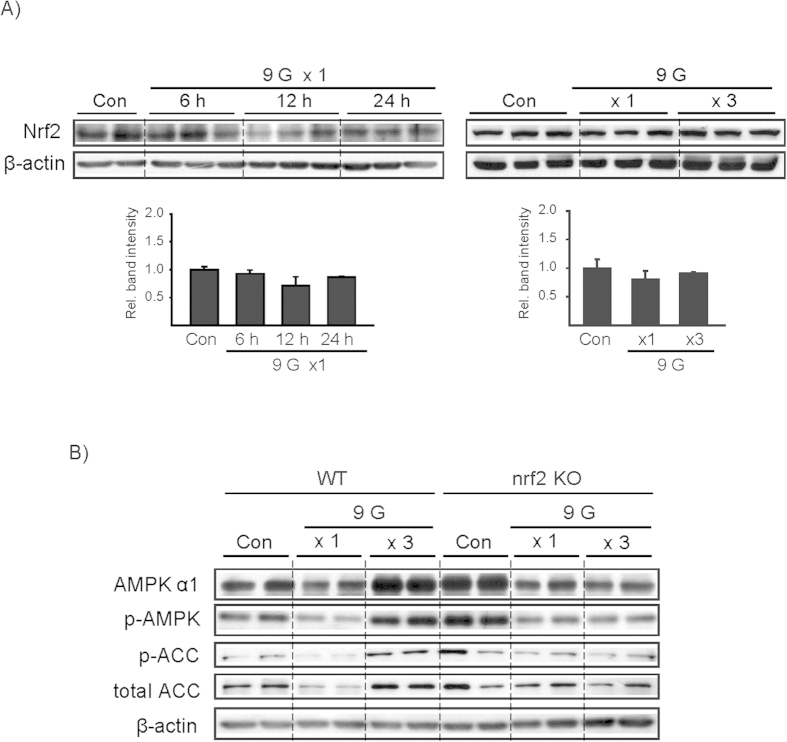
Nrf2 levels after load(s) to +9 Gz and the effect of Nrf2 gene knockout on AMPK. **(A**) Immunoblottings for Nrf2 in the liver. Mice were treated as described in the panels to Fig. 2. Of six different samples, two or three representative lanes were shown in each group. Data represent the mean±S.E (N = 6 each). No statistical significance of the differences was found between each treatment group and control. **(B)** Immunoblottings on the liver samples. C57BL/6 mice (wild type) or Nrf2 gene knockout (KO) mice were treated as described in the panel A to Fig. 3. Of three different samples, two lanes were shown in each group.

The differential effect of a single or multiple load(s) to +9 Gz on Nrf2-dependent and Nrf2-independent genes prompted us to explore the link between Nrf2 and adaptive recovery of AMPKα. In immunoblotting assays, Nrf2 levels were not significantly changed during the resting times after a load to hypergravity (Fig. 4A). In addition, multiple exposures to +9 Gz did not change Nrf2 levels. Next, we used Nrf2 knockout (KO) animals to assess the impact of Nrf2 on the adaptive change of AMPKα. Intriguingly, a deficiency of Nrf2 completely abrogated restoration of AMPKα in the liver of mice loaded to +9 Gz hypergravity 3 times (Fig. 4B). This was verified by AMPKα or ACC phosphorylation. Our results indicate that Nrf2 may be necessary for adaptive recovery of AMPKα and the signaling pathway in the liver against hypergravity stress.

### Liver injury markers after +9 Gz hypergravity load

**Figure 5:**
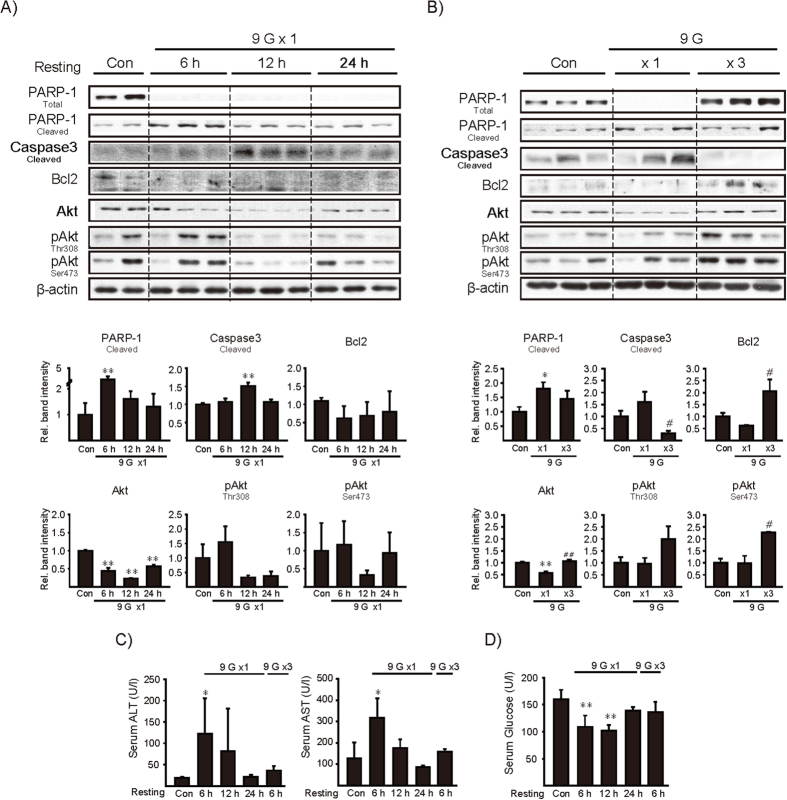
The effects of +9 Gz load(s) on the markers associated with hepatocyte viability or blood glucose contents. **(A**,**B**) Immunoblottings for the molecules associated with apoptosis or cell survival in the liver. Mice were treated as described in the panels to Fig. 2. Of six different samples, two or three representative lanes were shown in each group. **(C)** Serum transaminase activities. **(D)** Serum glucose levels. For A-D, data represent the mean±S.E (N = 6 each). Statistical significance of the differences was determined as compared to control group (*P < 0.05, **P < 0.01) or to a single load to +9 Gz (^#^*P* < 0.05, ^##^*P* < 0.01).

In subsequent experiments, we determined whether +9 Gz hypergravity load deteriorates hepatocyte function by measuring apoptosis or cell-survival markers. Total PARP-1 contents were markedly diminished with reciprocal increases of cleaved PARP-1 throughout the times examined (Fig. 5A upper). Of the time points, cleaved PARP-1 was most distinctly increased 6 h after a single load to +9 Gz. Consistently, cleaved caspase-3, a terminal caspase indicating apoptosis, was greatly elevated at 12 h. Likewise, Bcl2 levels were decreased. Total Akt was lowered 6 h or later times with variations noted in p-Akt levels (Ser473/Thr308). Repeated experiments confirmed alterations in apoptosis or cell-survival markers although variations existed in the animal experimentation (Fig. 5A lower). Adaptation to repeated hypergravity loads was also verified by molecular markers (Fig. 5B).

To confirm hepatocyte injury, we next examined the time-course profile of transaminases in the blood during the resting period. ALT and AST activities were increased 6 h after a single load to +9 Gz and then gradually returned toward normal at 24 h (Fig. 5C). Increases of ALT and AST activities were not found in the animals exposed to 3 consecutive loads, confirming that training exposures induce adaptation. Blood glucose levels were 32%–37% decreased 6 or 12 h after a +9 Gz hypergravity load, and returned to normal range at 24 h (Fig. 5D), being consistent with the degrees of liver dysfunction and inhibition of gluconeogenesis. Repetitive training loads also overcame hypoglycemia. Overall, our results provide strong evidence that a load to +9 Gz triggers apoptotic cascade, decreasing hepatocyte survival, and which can be overcome by preconditioning loads to hypergravity.

### Transcription factors and mitochondrial markers after a single or multiple +9 Gz load(s)

**Figure 6:**
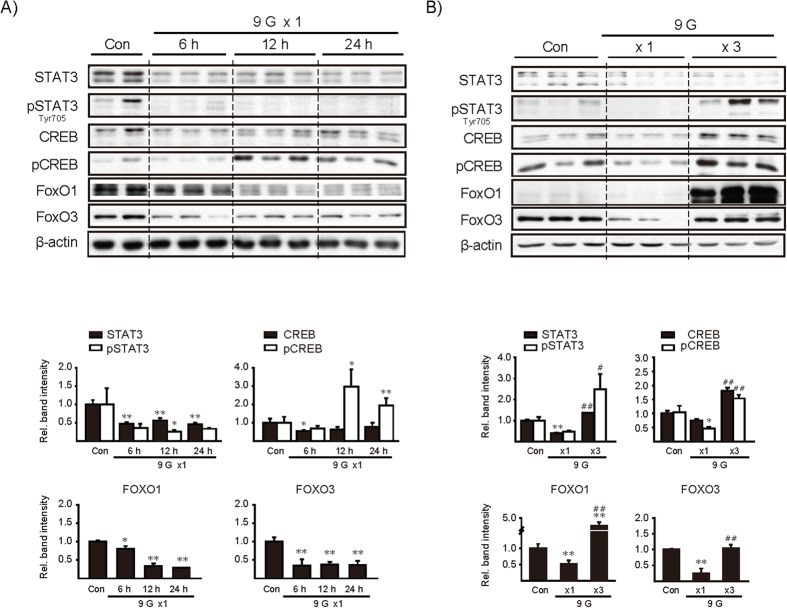
The effects of +9 Gz load(s) on hepatic transcription factors. **(A,B**) Immunoblottings for major transcription factors in the liver. Mice were treated as described in the panels to Fig. 2. Of six different samples, two or three representative lanes were shown in each group. Data represent the mean±S.E (N = 6 each). Statistical significance of the differences was determined as compared to control group (*P < 0.05, **P < 0.01) or to a single load to +9 Gz (^#^*P* < 0.05, ^##^*P* < 0.01). Con, control.

We then determined whether single or multiple load(s) to +9 Gz alter(s) transcription factor levels in the liver. In the assays, STAT3, CREB, FOXO1 and FOXO3 were included because they are associated with IL-6 and insulin/IGF-1/PI3K pathways^32^,^33^. A single load to +9 Gz mostly repressed expression of the transcription factors and/or their phosphorylation levels (Fig. 6A). They were recovered or rather enhanced 6 h after last exposure to +9 Gz in the multiple load model despite some variations (Fig. 6B). These results showed that most of the transcription factors examined were inhibited by a load of hypergravity, and the expression levels were restored or enhanced after repetitive loads.

**Figure 7:**
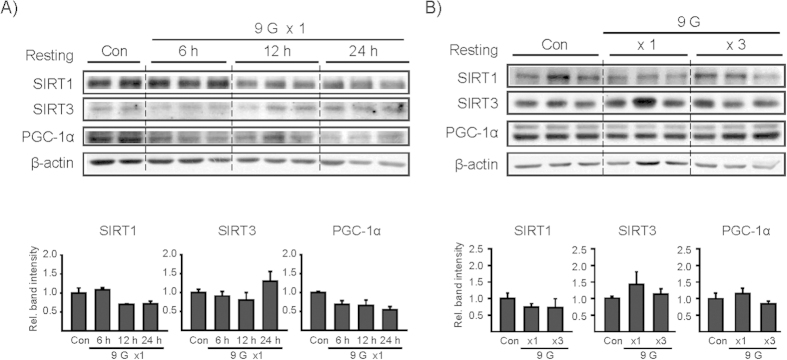
The effects of +9 Gz load(s) on SIRT and PGC-1α levels. **(A,B**) Immunoblottings for SIRT1, SIRT3, and PGC-1α. Mice were treated as described in the panels to Fig. 2. Of six different samples, two or three representative lanes were shown in each group. Data represent the mean±S.E (N = 6 each). No statistical significance of the differences was found between each treatment group and control. Con, control.

Since +9 Gz hypergravity stress consistently and significantly repressed AMPKα in the liver, we additionally determined SIRTs and peroxisomal proliferators-activated receptor γ coactivator (PGC-1α) as the indicators of mitochondrial oxidative function and fuel utilization. SIRT1, SIRT3 and PGC-1α levels failed to change consistently after a single or multiple load(s) to +9 Gz (Fig. 7A,B). This was confirmed by repeated experiments.

### Restraint effect on the molecular markers affected by +9 Gz hypergravity

**Figure 8:**
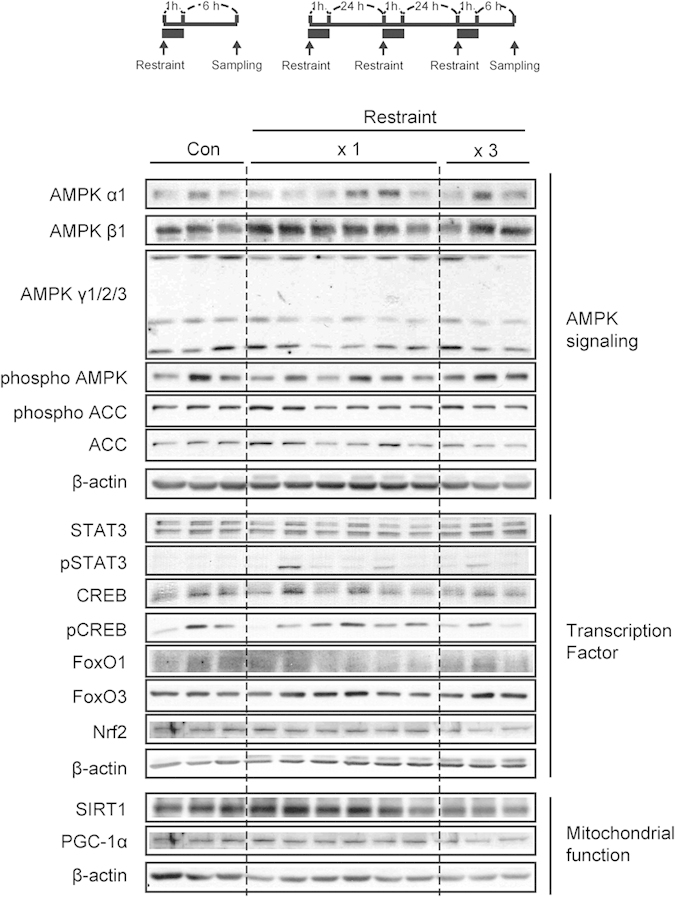
Immunoblottings for hepatic molecules in mice subjected to restraint stress. C57BL/6 mice were exposed to either a single restraint stress (x1) or three consecutive daily stresses (x3), and the liver samples were obtained 6 h after treatment. Of six different samples, three or six representative lanes were shown in each group. Con, control.

When a manned spacecraft is exposed to hypergravity stress, astronauts or jetfighters are restrained in position. So, we further assessed the effect of restraint on the parameters examined in the hypergravity model; mice were placed inside a cylindrical plastic restraint device for 1 h and then subjected to resting for 6 h. In another set, mice were placed in a similar restraint stress for 3 consecutive days. We found that restraint alone did not notably change AMPKα or other molecules of our interest (Fig. 8). In the analyses of transcription factors, STAT3, CREB, FOXOs and Nrf2 levels were also unaffected. In addition, there were no changes in SIRT1 and PGC-1α. Our results support the notion that a single or multiple restraint(s) of mice (1 h each) has no measurable effect on the levels of AMPKα and other markers affected by +9 Gz hypergravity load(s).

## Methods

### Experimental animals

Male C57BL/6 mice (8 weeks old) were purchased from Charles River Orient (Seoul, Korea), and housed at 20 ± 2 °C with 12-h light/dark cycles and a relative humidity of 50 ± 5% (Tecniplast, Varese, Italy) under filtered, pathogen-free air, with food (Purina, Korea) and water available *ad libitum*. Mice were acclimatized for 1 week at the Animal Center for Pharmaceutical Research, Seoul National University. Nrf2 KO mice supplied by RIKEN BioResource Center (Tsukuba, Japan) were bred and maintained. Nrf2 KO C57/BL6 mice were backcrossed with WT C57/BL6 mice for at least 6 months, and only male mice were used for experiments. Moreover, the protocol was approved by the local ethics committee (authorization number SNU-141103-1). Animal experiments were conducted under the guidelines and ethical approvals of the Institutional Animal Use and Care Committee at Seoul National University.

### Centrifugation experiment

The mice were exposed to short-term hypergravity at +9 Gz for 1 h using the small animal centrifuge at the Aerospace Medicine Research Center (Cheongju, Korea). The mice were placed inside a cylindrical plastic restraint device that, when mounted in the centrifuge, allowed +Gz to be delivered along the rostrocaudal axis. Once the mice were secured, the restraint device was placed onto the centrifuge. A cage-mounting module was attached at the end of the arm that allowed for one degree of freedom, thereby ensuring that the net gravity field was perpendicular to the floor of the restraint device. The mice were sacrificed at 6, 12 or 24 h after exposure to the indicated hypergravity. In different sets, animals were daily loaded to hypergravity for three days and the tissue samples were obtained 6 h after last load (Fig. 2A). The behavior of mice was monitored with a charge-coupled device camera throughout the centrifugation experiments.

### Blood chemistry

Plasma alanine aminotransferase (ALT) activity, aspartate aminotransferase (AST) and glucose contents were analyzed using Spectrum, an automatic blood chemistry analyzer (Abbott Laboratories, Abbott Park, IL, USA).

### Hematoxylin and eosin staining

The left lateral lobe of the liver was sliced, and tissue slices were fixed in 10% buffered-neutral formalin for 6 h. The samples were stained with hematoxylin and eosin (H&E).

### Immunoblot analysis

Proteins were separated by 7.5% sodium dodecyl sulfate polyacrylamide gel electrophoresis and transferred onto nitrocellulose membrane (Millipore, Bedford, MA). The membrane was blocked with 5% non-fat dried milk in TBST (20 mM Tris-HCl, 150 mM NaCl, and 0.1% Tween 20, pH 7.5) for 1 h, and incubated overnight with each primary antibody at 4 °C. After washing with TBST buffer, membranes were incubated with secondary antibodies for 1 h at room temperature. The protein bands were visualized using an ECL chemiluminescence system (Amersham, Buckinghamshire, UK). Equal loading of samples was verified by immunoblotting for β-actin. Band intensities were quantified using Adobe Photoshop CS5 (Adobe Systems, San Jose, CA).

### RNA quality check

For the quality control, RNA purity and integrity were evaluated by OD 260/280 ratio, and analyzed by Agilent 2100 Bioanalyzer (Agilent Technologies, Palo Alto, USA).

### Affymetrix whole transcript expression arrays methods

The Affymetrix Whole Transcript Expression array process was executed according to the manufacturer’s protocol (GeneChip Whole Transcript PLUS reagent Kit). cDNA was synthesized using the GeneChip WT Amplification kit as described by the manufacturer. The sense cDNA was then fragmented and biotin-labeled with TdT (terminal deoxynucleotidyl transferase) using the GeneChip WT Terminal labeling kit. Approximately 5.5 μg of labeled DNA target was hybridized to the Affymetrix GeneChip Mouse 2.0 ST Array at 45 °C for 16 h. Hybridized arrays were washed and stained on a GeneChip Fluidics Station 450 and scanned on a GCS3000 Scanner (Affymetrix).

### Raw data preparation and statistical analysis

Raw data were extracted automatically in Affymetrix data extraction protocol using the software provided by Affymetrix GeneChip® Command Console® Software (AGCC). After importing CEL files, the data were summarized and normalized with robust multi-average (RMA) method implemented in Affymetrix® Expression Console™ Software (EC). We exported the result with gene level RMA analysis and performed the differentially expressed gene (DEG) analysis. The comparative analyses between test samples and control samples were carried out using LPE test and fold changes. False discovery rate (FDR) was controlled by adjusting p value using Benjamini-Hochberg algorithm. For a DEG set, hierarchical cluster analysis was done using complete linkage and Euclidean distance as a measure of similarity. All statistical test and visualization of differentially expressed genes was conducted using R statistical language v. 3.0.2. (www.r-project.org).

### Data analyses

For animal experiments, data represent the mean ± S.E. The differences between groups were analyzed using one-tailed Student’s t-test. The criterion for statistical significance was set at *P* < 0.05 or *P* < 0.01.

## Discussion

An understanding of the biological responses to hypergravity is important to prevent unwanted responses that occur during exposure to hypergravity. In this study, we evaluated the effect of +Gz accelerative gravity, a force applied to the vertical axis from head to foot, on the body in animal models. A new equation created using the reported effects of gravity on mortality vs. masses of animal species enabled us to estimate gravity load in mice which can be extrapolated to humans. Our data obtained from +3 or +9 Gz load seems to reflect the gravity force frequently attained in human situations. We found that hypergravity stress caused injuries in the skeletal muscle or kidney immediately after +3 or +9 Gz load. However, the parameters returned toward normal during resting times. In contrast, +9 Gz hypergravity stress caused marked changes in the liver during the resting time, implying that the liver tissue may be vulnerable to physical or compressional stress progressively.

Alteration of gravity may enhance compressional stress, accompanying disturbances in biological responses and increase of oxidative stress. The liver is a continuous sponge-like parenchymal mass consisting of the interdigitating networks of afferent and efferent vessels; the hepatic vasculature, particularly sinusoids comprising 60% of the liver vascular volume, is featured by high compliance, high capacity and energy metabolism^34^. In our findings, a load to +9 Gz hypergravity severely affected the levels of molecules associated with energy metabolism, altering blood biochemical indexes representing liver injury. Likewise, a pressure stress elicited by hyper-gravitational force increased apoptotic marker levels in the liver. Serum aminotransferase activities worsened at later time after the compressional stress, indicative of delayed injury of hepatocytes to hypergravity load and intrinsic vulnerability of the liver tissue. Hepatocytes stressed metabolically or hemodynamically (e.g., aged cells) would be more susceptible to hypergravity presumably due to energy shortage and/or hypoxia, which remains to be clarified in the future.

In the hepatic cell, AMPK serves as a sensor for energy homeostasis, responding to the change in the AMP:ATP ratio^35^. AMPK activation then leads to threonine-172 phosphorylation in the catalytic domain of α subunit, promoting fuel utilization with the inhibition of gluconeogenesis and of glycogen, fatty acid and cholesterol synthesis. Our data that a load to +9 Gz hypergravity significantly repressed AMPKα subunit (a catalytic subunit α1/2) 6 h post-treatment represents progressive metabolic disturbances in hepatocytes. A sufficient resting time (i.e., 24 h or longer time) seems to be required for full recovery of hepatocytes from compressional stress. This contention is further supported by the finding that p-AMPK and p-ACC levels were initially lowered and gradually returned toward basal during the resting period. Another important finding of this study is that repetitive exposures of mice to +9 Gz for three days could induce an adaptive capability to overcome hypergravity stress. Moreover, our result supports the concept that recovery of energy-metabolizing capacity may be indispensable for adaptation to hypergravity stress. This was also reflected by the changes in p-AMPK and p-ACC.

The cDNA microarray analyses yielded new and interesting information as to the complex and interrelated biological processes. Of the clusters of genes affected by hypergravity, we placed an emphasis on Nrf2 and its target gene expression, and found that the expression of Nrf2 target genes was repressed 6 h after a load to hypergravity despite no notable change in Nrf2 level, raising the concept that hypergravity stress causes oxidative stress. Moreover, our data support the idea that Nrf2 plays a role in defending the cell from hypergravity-induced compressional stress in association with the recovery of antioxidant capacity. An intriguing finding of our study is that a deficiency of Nrf2 abrogated the adaptive increase of AMPKα elicited by repetitive loads to hypergravity, implying that Nrf2 plays a role in AMPK-dependent ATP production under the condition of hypergravity stress. In a separate study, we discovered the ability of AMPK to regulate Nrf2 trafficking to the nucleus (Joo and Kim *et al.*, manuscript submitted). This event along with the present findings strongly supports the close association between AMPK signaling and Nrf2-target gene expression.

Studies had been done on the effects of microgravity on animals and cells. Exposure of rats to +1.65 G hypergravity from gestational day 8 to 21 may cause oxidative stress on the developing brain^36^. On the other hand, chronic microgravity exposure to simulated space conditions alters oxidative stress response in mouse fetal fibroblast^37^. In rat cerebral arteries, microgravity causes mitochondrial dysfunction with increase of mitochondrial reactive oxygen species, and mitochondria-targeted antioxidant promoted recovery of mitochondrial function^38^. Our finding showed that STAT3, CREB and FoxOs (i.e., transcription factors associated with cell survival and cell cycle control) were markedly inhibited by a load to +9 Gz hypergravity (e.g., production of acute-phase proteins in hepatocytes by IL-6; gp130-IL-6 R regulation of STAT3; FoxO1 regulation by IGF-1/PI3K pathway; FoxO3 control of cell cycle arrest and apoptosis; and CREB regulation of FoxO1), and the changes were minimized or abrogated after multiple loads of hypergravity. Of the transcription factors, FoxO1 affects a variety of physiological events including cell proliferation, antioxidant capacity and adaptive stress responses^39^,^40^,^41^. FoxO1 also controls hepatic gluconeogenesis^42^, sustaining the normal glucose content. FoxO1 levels changed by hypergravity well reflect stress and adaptive responses. SIRT1/3 may control mitochondrial fuel oxidation and lipid homeostasis in association with PGC-1α^43^,^44^. Our finding that +9 Gz hypergravity load(s) had a minimal effect on SIRT1/3 and PGC-1α supports the conclusion that hypergravity-induced compressional stress preferentially alters AMPKα level and the signaling pathway.

In conclusion, we showed that a load to +9 Gz hypergravity immediately induced compressional stress on the organs including the liver, skeletal muscle, and kidney, and that this stress progressively caused decreases of AMPKα level, the downstream energy production pathway, and Nrf2-dependent antioxidant gene expression in the liver, presumably deteriorating ATP production and worsening compressional injury of hepatocytes. In addition, our study demonstrated that repetitive preconditioning loads enhanced the adaptive capability of mice to hypergravity stress, as mediated in part by Nrf2-dependent recovery of AMPK pathway.

## Additional Information

**How to cite this article**: Lee, S.G. *et al.* A load of mice to hypergravity causes AMPKα repression with liver injury, which is overcome by preconditioning loads via Nrf2. *Sci. Rep.* **5**, 15643; doi: 10.1038/srep15643 (2015).
