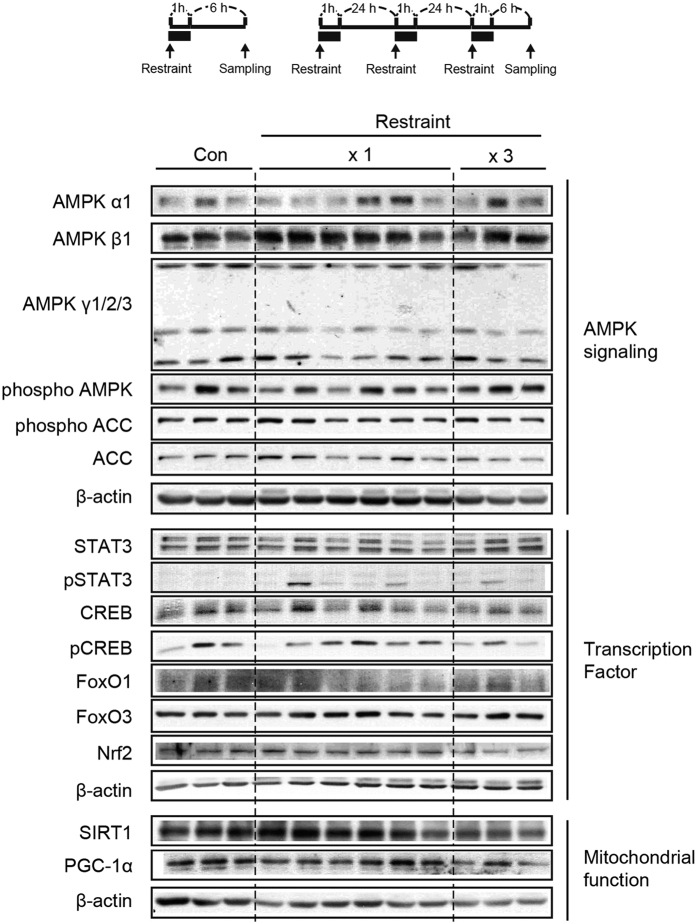# Corrigendum: A load of mice to hypergravity causes AMPKα repression with liver injury, which is overcome by preconditioning loads via Nrf2

**DOI:** 10.1038/srep34677

**Published:** 2016-10-05

**Authors:** Sang Gil Lee, Chan Gyu Lee, Hong Min Wu, Choong Sik Oh, So Won Chung, Sang Geon Kim

Scientific Reports 5: Article number: 1564310.1038/srep15643; published online: 10 23 2015; updated: 10 05 2016

This Article contains errors in Figure 5A and Figure 8.

In Figure 5A, the immunoblot for ‘Akt’ is incorrect.

In Figure 8, the immunoblot for Nrf2 is incorrectly duplicated as PGC-1α.

The correct Figures 5A and 8 appear below as Figure 1 and Figure 2 respectively.

## Figures and Tables

**Figure 1:**
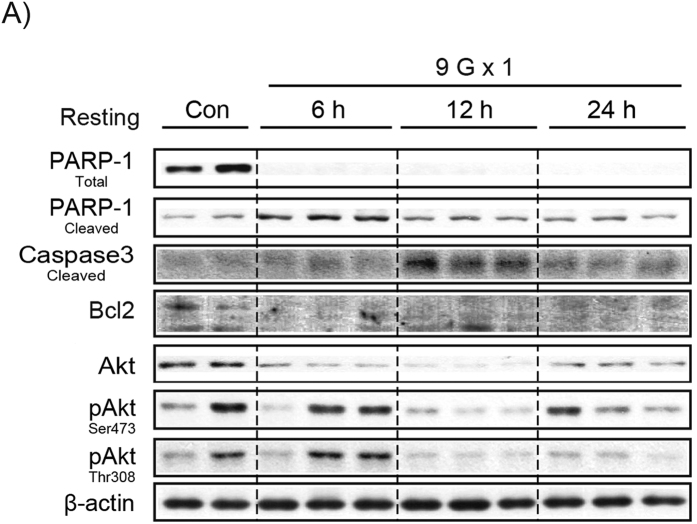


**Figure 2:**